# Modified Fluorouracil, Leucovorin, Irinotecan, and Oxaliplatin or S-1, Irinotecan, and Oxaliplatin Versus Nab-Paclitaxel + Gemcitabine in Metastatic or Recurrent Pancreatic Cancer (GENERATE, JCOG1611): A Randomized, Open-Label, Phase II/III Trial

**DOI:** 10.1200/JCO.24.00936

**Published:** 2025-07-28

**Authors:** Akihiro Ohba, Masato Ozaka, Junki Mizusawa, Takuji Okusaka, Satoshi Kobayashi, Taro Yamashita, Masafumi Ikeda, Ichiro Yasuda, Kazuya Sugimori, Naoki Sasahira, Kenji Ikezawa, Ikuya Miki, Naohiro Okano, Nobumasa Mizuno, Masayuki Furukawa, Hirofumi Shirakawa, Yusuke Sano, Hiroshi Katayama, Junji Furuse, Makoto Ueno, on behalf of the Hepatobiliary and Pancreatic Oncology Group of the Japan Clinical Oncology Group (JCOG-HBPOG), Keiya Okamura

**Affiliations:** ^1^ Division of Gastrointestinal Oncology, Shizuoka Cancer Center, Shizuoka, Japan; ^2^ Department of Hepato-Biliary-Pancreatic Medicine, Cancer Institute Hospital of Japanese Foundation for Cancer Research, Tokyo, Japan; ^3^ JCOG Data Center, National Cancer Center Hospital, Tokyo, Japan; ^4^ Department of Hepatobiliary and Pancreatic Oncology, National Cancer Center Hospital, Chuo, Japan; ^5^ Department of Gastroenterology, Kanagawa Cancer Center, Yokohama, Japan; ^6^ Department of Gastroenterology, Graduate School of Medical Sciences, Kanazawa University, Kanazawa, Japan; ^7^ Department of Hepatobiliary and Pancreatic Oncology, National Cancer Center Hospital East, Kashiwa, Japan; ^8^ Third Department of Internal Medicine, University of Toyama, Toyama, Japan; ^9^ Gastroenterological Center, Yokohama City University Medical Center, Yokohama, Japan; ^10^ Department of Hepatobiliary and Pancreatic Oncology, Osaka International Cancer Institute, Osaka, Japan; ^11^ Department of Gastroenterology, Hyogo Cancer Center, Akashi, Japan; ^12^ Department of Medical Oncology, Kyorin University Faculty of Medicine, Mitaka, Japan; ^13^ Department of Gastroenterology, Aichi Cancer Center Hospital, Nagoya, Japan; ^14^ Department of Hepato-Biliary-Pancreatology, National Hospital Organization, Kyushu Cancer Center, Fukuoka, Japan; ^15^ Department of Hepato-Biliary-Pancreatic Surgery, Tochigi Cancer Center, Utsunomiya, Japan; ^16^ JCOG Operations Office, National Cancer Center Hospital, Chuo, Japan

## Abstract

**PURPOSE:**

Modified fluorouracil, leucovorin, irinotecan, and oxaliplatin (mFOLFIRINOX) and nab-paclitaxel + gemcitabine are recommended as first-line treatments for metastatic pancreatic cancer. S-1, irinotecan, and oxaliplatin (S-IROX) demonstrated activity in a phase Ib trial in this population. Therefore, these three regimens were directly compared.

**METHODS:**

This randomized phase II/III trial was performed at 45 centers in Japan. Eligible patients age 20-75 years with an Eastern Cooperative Oncology Group performance status of 0 or 1 and pathologically confirmed metastatic or recurrent pancreatic cancer were randomly assigned (1:1:1) to receive mFOLFIRINOX (oxaliplatin 85 mg/m^2^ over 2 hours, irinotecan 150 mg/m^2^ over 90 minutes, l-leucovorin 200 mg/m^2^ over 2 hours, each once daily on day 1, and fluorouracil 2,400 mg/m^2^ over 46 hours on days 1-3, every 2 weeks), S-IROX (oxaliplatin 85 mg/m^2^ over 2 hours, irinotecan 150 mg/m^2^ over 90 minutes on day 1, and S-1 80 mg/m^2^/day administered orally twice daily on days 1-7, every 2 weeks), or nab-paclitaxel (125 mg/m^2^) + gemcitabine (1,000 mg/m^2^) on days 1, 8, and 15 every 4 weeks. The primary end point was overall survival (OS).

**RESULTS:**

A total of 527 patients were enrolled, with 426 included in the planned interim analysis. The median OS was 14.0 months (hazard ratio [HR], 1.31 [95% CI, 0.97 to 1.77]) and 13.6 months (HR, 1.35 [95% CI, 1.00 to 1.82]) in the mFOLFIRINOX and S-IROX groups, respectively, as compared with 17.1 months in the nab-paclitaxel + gemcitabine group. The predictive probability of achieving superiority in the final analysis was <1% in both groups. Thus, the trial was terminated owing to its futility. Grade 3 to 4 anorexia was more frequent in the mFOLFIRINOX (23.3%) and S-IROX (27.5%) groups than in the nab-paclitaxel + gemcitabine group (5.0%).

**CONCLUSION:**

Neither mFOLFIRINOX nor S-IROX appeared to be superior compared with nab-paclitaxel + gemcitabine as the first-line treatment for metastatic or recurrent pancreatic cancer.

## INTRODUCTION

Pancreatic cancer is a leading cause of cancer-related deaths worldwide.^1^ More than 80% of patients have unresectable or metastatic disease at the time of diagnosis, and the 5-year overall survival (OS) is ≤10%.^2^ Systemic chemotherapy is the standard of care for patients with metastatic pancreatic cancer because immune checkpoint inhibitors or molecularly targeted agents have limited benefits. Fluorouracil, leucovorin, irinotecan, and oxaliplatin (FOLFIRINOX; including modified FOLFIRINOX [mFOLFIRINOX]) and nab-paclitaxel + gemcitabine are recommended as first-line treatments for metastatic pancreatic cancer, with good performance status across various guidelines.^3,4^ Although fluorouracil, leucovorin, liposomal irinotecan, and oxaliplatin (NALIRIFOX) is superior to nab-paclitaxel + gemcitabine,^5^ there are no direct comparisons between these two regimens, raising questions concerning the optimal regimen and its appropriate use.

**CONTEXT**

**Key Objective**
Both modified fluorouracil, leucovorin, irinotecan, and oxaliplatin (mFOLFIRINOX) and nab-paclitaxel + gemcitabine are recommended first-line treatments for metastatic pancreatic cancer; however, no direct comparison has been conducted. The GENERATE (JCOG1611) trial, a phase II/3 study, directly compared these two treatments, along with S-1, irinotecan, and oxaliplatin (S-IROX).
**Knowledge Generated**
In 527 Japanese patients, neither mFOLFIRINOX nor S-IROX demonstrated superiority in terms of overall survival (OS) over nab-paclitaxel + gemcitabine. OS was numerically longer with nab-paclitaxel + gemcitabine, whereas GI toxicity was more frequently observed with mFOLFIRINOX and S-IROX.
**Relevance *(E.M. O'Reilly)***
This study provides continued support for doublet regimens (gemcitabine/nab-paclitaxel) for untreated metastatic pancreatic cancer with a good performance status; however, the field clearly needs to move beyond chemotherapy alone and biomarker based selection is urgently needed in this disease.*
*Relevance section written by *JCO* Associate Editor Eileen M. O'Reilly, MD, FASCO.

The PRODIGE 4/ACCORD 11 trial established the survival benefit of the original FOLFIRINOX regimen over that of gemcitabine monotherapy in patients with metastatic pancreatic cancer.^6^ However, a Japanese phase II trial of this regimen revealed frequent hematologic toxicities.^7^ An mFOLFIRINOX regimen, without bolus fluorouracil and with a reduced dose of irinotecan (150 mg/m^2^ once every 2 weeks), demonstrated good safety and efficacy in patients with metastatic pancreatic cancer,^8^ leading to widespread clinical adoption. The MPACT trial reported that nab-paclitaxel + gemcitabine was superior to gemcitabine monotherapy in patients with metastatic pancreatic cancer.^9^ Subsequently, a phase I/II trial of nab-paclitaxel + gemcitabine confirmed similar efficacy and safety in Japanese patients,^10^ and this regimen was widely accepted. We hypothesized that mFOLFIRINOX was more promising in terms of OS because of the lower hazard ratio (HR) for gemcitabine monotherapy in the phase III trial. S-1, an oral fluoropyrimidine noninferior to gemcitabine monotherapy in a phase III trial,^11^ was a standard-of-care option before combination chemotherapy emerged. S-1, irinotecan, and oxaliplatin (S-IROX) was expected to improve efficacy and patient convenience by eliminating continuous intravenous infusion of fluorouracil. In a phase Ib trial, S-IROX demonstrated promising antitumor activity with a good safety profile.^12^

Consequently, the randomized phase II/III GENERATE (JCOG1611) trial aimed to confirm the superiority of mFOLFIRINOX or S-IROX over nab-paclitaxel + gemcitabine as a first-line treatment in patients with metastatic or recurrent pancreatic cancer.

## METHODS

### Trial Oversight

The trial adheres to the international ethical recommendations outlined in the Declaration of Helsinki and the Clinical Trials Act in Japan. The study protocol was approved by the Japan Clinical Oncology Group (JCOG; Data Supplement, online only) Protocol Review Committee and the National Cancer Center Hospital Certified Review Board. The JCOG Data and Safety Monitoring Committee (DSMC) oversaw the trial data and operations. This trial was registered with the Japan Registry of Clinical Trials (jRCTs031190009).

### Patients

Eligible patients were age 20-75 years and had an Eastern Cooperative Oncology Group performance status (ECOG PS) of 0 or 1, with histologically or cytologically confirmed pancreatic ductal adenocarcinoma or adenosquamous carcinoma that was previously untreated and metastatic or recurrent. Patients who underwent chemotherapy or chemoradiotherapy for pancreatic cancer were ineligible, except for those who had received their last dose of S-1 or gemcitabine as adjuvant chemotherapy for ≥24 weeks before trial enrollment and received gemcitabine + S-1 as neoadjuvant chemotherapy. Patients were required to have wild-type (–/–) or single heterozygous (*6/– or *28/–) uridine diphosphate glucuronosyltransferase 1A1 (UGT1A1) genotypes, sufficient oral intake of food, and adequate organ functions. The full eligibility criteria are provided in the Data Supplement. All patients provided written informed consent before trial enrollment.

### Study Design

GENERATE was a multicenter, open-label, randomized phase II/III trial conducted at 45 Japanese academic centers participating in the Hepatobiliary and Pancreatic Oncology Group of the JCOG. The patients were randomly assigned (1:1:1) to receive nab-paclitaxel + gemcitabine, mFOLFIRINOX, or S-IROX. Random assignment was performed using the minimization method with a random component adjusted for stratification factors of the institution, ECOG PS (0 *v* 1), and the disease status (metastatic *v* recurrent).

### Treatment

Patients were intravenously administered 125 mg/m^2^ nab-paclitaxel + 1,000 mg/m^2^ gemcitabine on days 1, 8, and 15, every 4 weeks; mFOLFIRINOX (oxaliplatin 85 mg/m^2^ over 2 hours, irinotecan 150mg/m^2^ over 90 minutes, l-leucovorin 200 mg/m^2^ over 2 hours, each once daily on day 1, and fluorouracil 2,400 mg/m^2^ over 46 hours on days 1-3, every 2 weeks); or S-IROX (oxaliplatin 85 mg/m^2^ over 2 hours, irinotecan 150mg/m^2^ over 90 minutes on day 1, and S-1 80 mg/m^2^/day administered orally twice daily on days 1-7, every 2 weeks). The protocol treatment was continued until radiologic (according to RECIST version 1.1) or clinical disease progression or unacceptable toxicity.

### Assessments and End Points

Tumor evaluation was performed using contrast-enhanced computed tomography or magnetic resonance imaging at baseline and every 6 weeks until disease progression or death in accordance with RECIST version 1.1. Physical and laboratory examinations were performed on treatment administration days during the protocol treatment.

The primary end point of the phase II portion was the objective response rate (ORR) in the S-IROX group. The primary end point of the phase III portion was OS in all randomly assigned patients. The secondary end points of the phase III portion were progression-free survival (PFS), ORR by local investigator review, adverse events graded by local investigators using the National Cancer Institute Common Terminology Criteria for Adverse Events version 4.0, and dose intensity.

### Statistical Analysis

This randomized phase II/III trial aimed to confirm the superiority of mFOLFIRINOX and S-IROX over nab-paclitaxel + gemcitabine in patients with metastatic or recurrent pancreatic cancer. For the phase II portion, the planned sample size was 45 patients in the S-IROX arm, with a one-sided alpha of 10%, a power of 90%, a threshold response rate of 20%, and an expected response rate of 40% based on the previous phase Ib trial with the response rate of 51.1%.^12^ For the phase III portion, the expected median OS was 11 months in the nab-paclitaxel + gemcitabine group and 15 months in the mFOLFIRINOX and S-IROX groups, corresponding to an HR of 0.73. The required sample size for the phase III portion for each comparison was 240 patients in each group (720 patients in total), considering a one-sided alpha of 1.25% and a power of 80% for each comparison.

One interim analysis of the phase III portion was planned after 50% of the planned number of patients was enrolled. The prespecified stopping criterion for the interim analysis was efficacy: if the OS of either mFOLFIRINOX or S-IROX was significantly superior to that of the nab-paclitaxel + gemcitabine group. However, if the survival curve for either mFOLFIRINOX or S-IROX was inferior to that of the nab-paclitaxel + gemcitabine group (ie, HR > 1.0), the corresponding experimental treatment group would be terminated because of futility considering various factors such as the extent of the magnitude of treatment effects and toxicities.

Regarding OS, the stratified log-rank test was used to test the superiority of mFOLFIRINOX to nab-paclitaxel + gemcitabine and S-IROX to nab-paclitaxel + gemcitabine, with a one-sided alpha of 1.25% in each comparison. Stratified Cox regression analyses were performed to estimate the HRs and CIs. The Kaplan-Meier method was used to estimate the time-to-event outcomes in each treatment group. PFS was analyzed using unstratified analyses. All subgroup analyses for efficacy were prespecified. All statistical analyses were conducted using the SAS version 9.4 software.

## RESULTS

### Planned Interim Analysis

**FIG 1.**
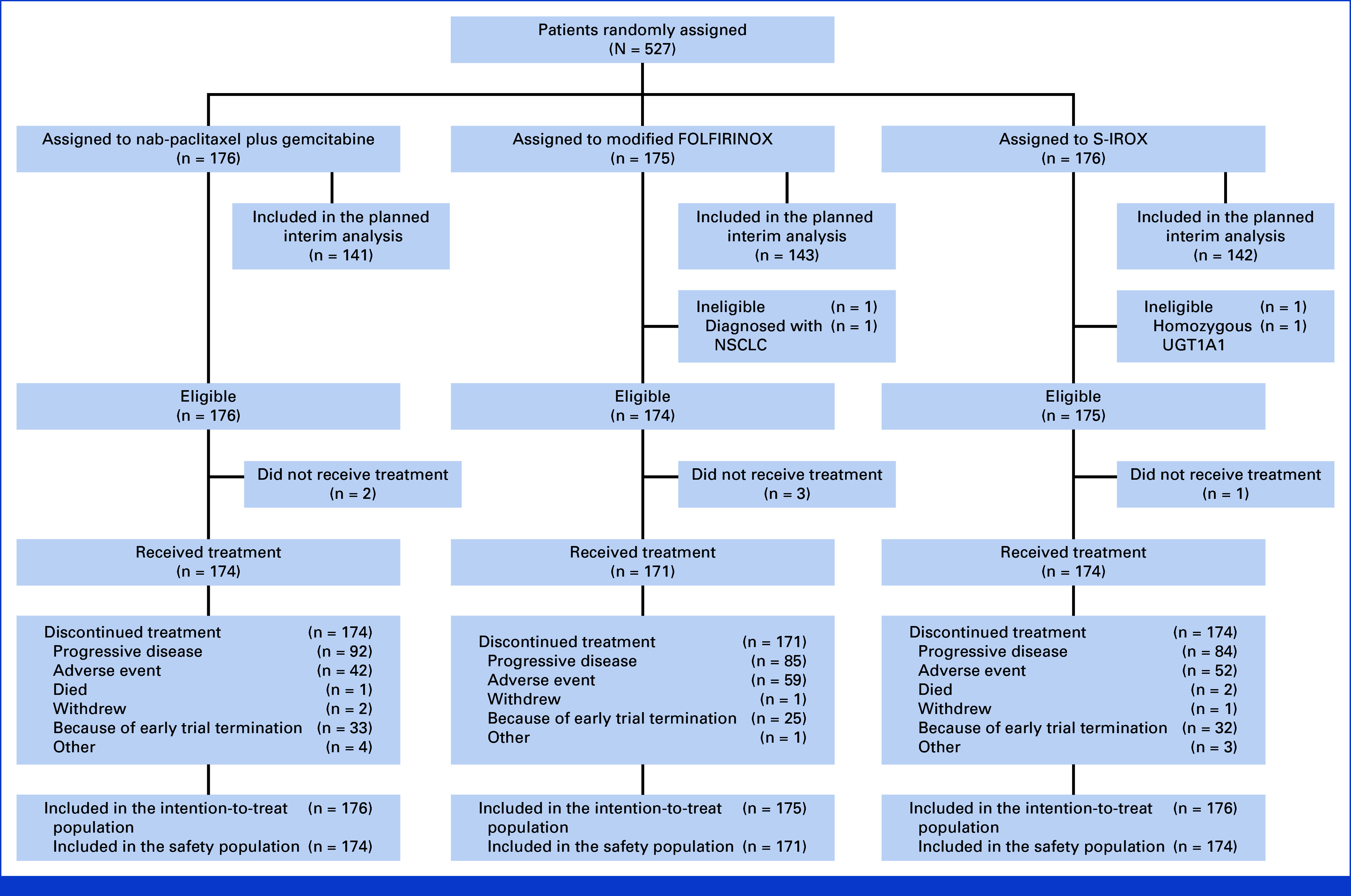
Trial profile. mFOLFIRINOX, modified fluorouracil, leucovorin, irinotecan, and oxaliplatin; NSCLC, non–small cell lung cancer; S-IROX, S-1, irinotecan, and oxaliplatin. UGT1A1, uridine diphosphate glucuronosyltransferase 1A1.

**FIG 2.**
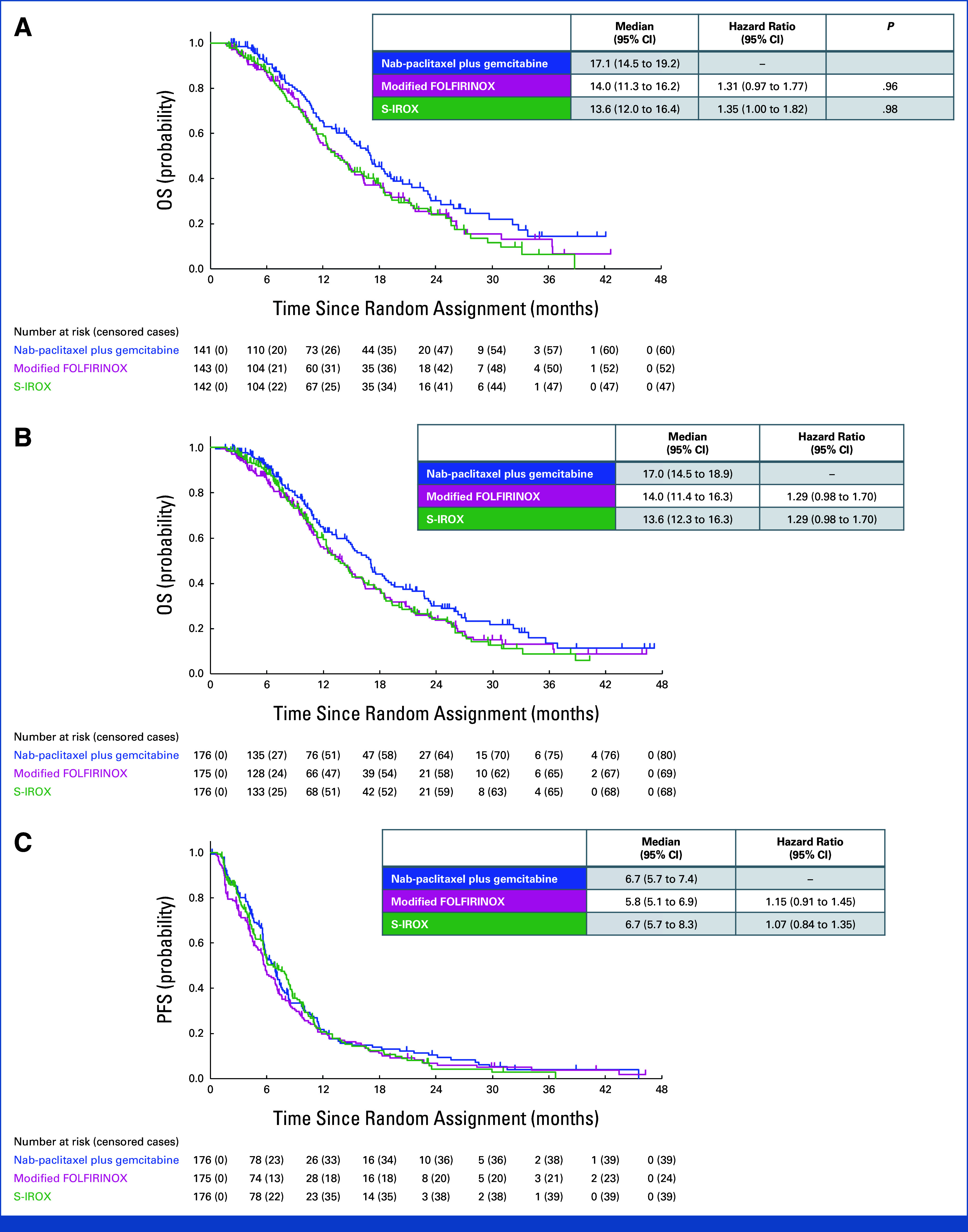
Kaplan-Meier estimates of (A) OS at the planned interim analysis, (B) updated OS, and (C) PFS. Tick marks indicate censored data. HRs are based on a stratified Cox regression model. HR, hazard ratio; mFOLFIRINOX, modified fluorouracil, leucovorin, irinotecan, and oxaliplatin; OS, overall survival; PFS, progression-free survival; S-IROX, S-1, irinotecan, and oxaliplatin.

Between Apr 2019 and Mar 2023, 527 patients were enrolled and randomly allocated to receive nab-paclitaxel + gemcitabine (n = 176), mFOLFIRINOX (n = 175), or S-IROX (n = 176; Fig 1, Table 1). Among these patients, 426 (nab-paclitaxel + gemcitabine, 141 patients; mFOLFIRINOX, 143 patients; S-IROX, 142 patients) were enrolled until September 2022 and included in the planned interim analysis. The median OS was 17.1 months (95% CI 14.5-19.2) in the nab-paclitaxel + gemcitabine group, 14.0 months (95% CI 11.3-16.2) in the mFOLFIRINOX group (HR, 1.31, 95% CI 0.97-1.77), and 13.6 months (95% CI 12.0-16.4) in the S-IROX group (HR, 1.35, 95% CI 1.00-1.82; Fig 2). The predictive probability of achieving superiority in the final analysis was < 1% for the mFOLFIRINOX and S-IROX groups, respectively. Furthermore, the conditional power was calculated to assess the likelihood of demonstrating statistical superiority at the final analysis, given the interim results and assuming that the HR for OS in both the mFOLFIRINOX and S-IROX groups versus the nab-paclitaxel plus gemcitabine group remained at the originally hypothesized value used in the sample size calculation (HR = 0.73). Under this assumption, the conditional power was estimated to be 1.4% for the mFOLFIRINOX group and <1% for the S-IROX group. Therefore, the JCOG DSMC recommended that the trial be terminated early because of futility, and the Hepatobiliary and Pancreatic Oncology Group of the JCOG accepted this recommendation.

**TABLE 1.** Baseline Characteristics (intention-to-treat population)

Characteristic	Nab-Paclitaxel + Gemcitabine (n = 176)	Modified FOLFIRINOX (n = 175)	S-IROX (n = 176)
Age, years, median (IQR, range)	65 (57-70, 38-75)	67 (59-71, 45-75)	65 (57-70, 35-75)
Sex			
Female	89 (50.6)	71 (40.6)	64 (36.4)
Male	87 (49.4)	104 (59.4)	112 (63.6)
ECOG PS			
0	118 (67.0)	118 (67.4)	119 (67.6)
1	58 (33.0)	57 (32.6)	57 (32.4)
Pathology			
Adenocarcinoma	171 (97.2)	171 (97.7)	173 (98.3)
Adenosquamous carcinoma	5 (2.8)	4 (2.3)	3 (1.7)
Disease status			
Metastatic	165 (93.8)	167 (95.4)	167 (94.9)
Recurrent	11 (6.3)	8 (4.6)	9 (5.1)
Metastatic sites			
1	116 (65.9)	103 (58.9)	109 (61.9)
2	44 (25.0)	51 (29.1)	50 (28.4)
≥3	16 (9.1)	21 (12.0)	17 (9.7)
Liver metastasis	117 (66.5)	124 (70.9)	124 (70.5)
Main pancreatic tumor location			
Head	68 (38.6)	68 (38.9)	70 (39.8)
Body or tail	108 (61.4)	107 (61.1)	106 (60.2)
Baseline CA 19-9			
<37 U/mL	33 (18.8)	39 (22.3)	28 (15.9)
≥37 U/mL	143 (81.2)	136 (77.7)	148 (84.1)

NOTE. Data are No. (%), unless otherwise stated.

Abbreviations: CA19-9, carbohydrate antigen 19-9; ECOG PS, Eastern Cooperative Oncology Group performance status; mFOLFIRINOX, modified fluorouracil, leucovorin, irinotecan, and oxaliplatin; S-IROX, S-1, irinotecan, and oxaliplatin.

### Patients

After random assignment, one patient in each of the mFOLFIRINOX and S-IROX groups was ineligible. Two patients in the nab-paclitaxel + gemcitabine group, three patients in the mFOLFIRINOX group, and one patient in the S-IROX group did not receive the protocol treatment. Thus, all 527 randomly assigned patients were included in the intention-to-treat (ITT) population, and the safety population consisted of 519 eligible patients. Germline mutation testing for *BRCA1* or *BRCA2* was performed in 249 (46.0%) of the 527 patients (Table S1).

### Efficacy

**FIG 3.**
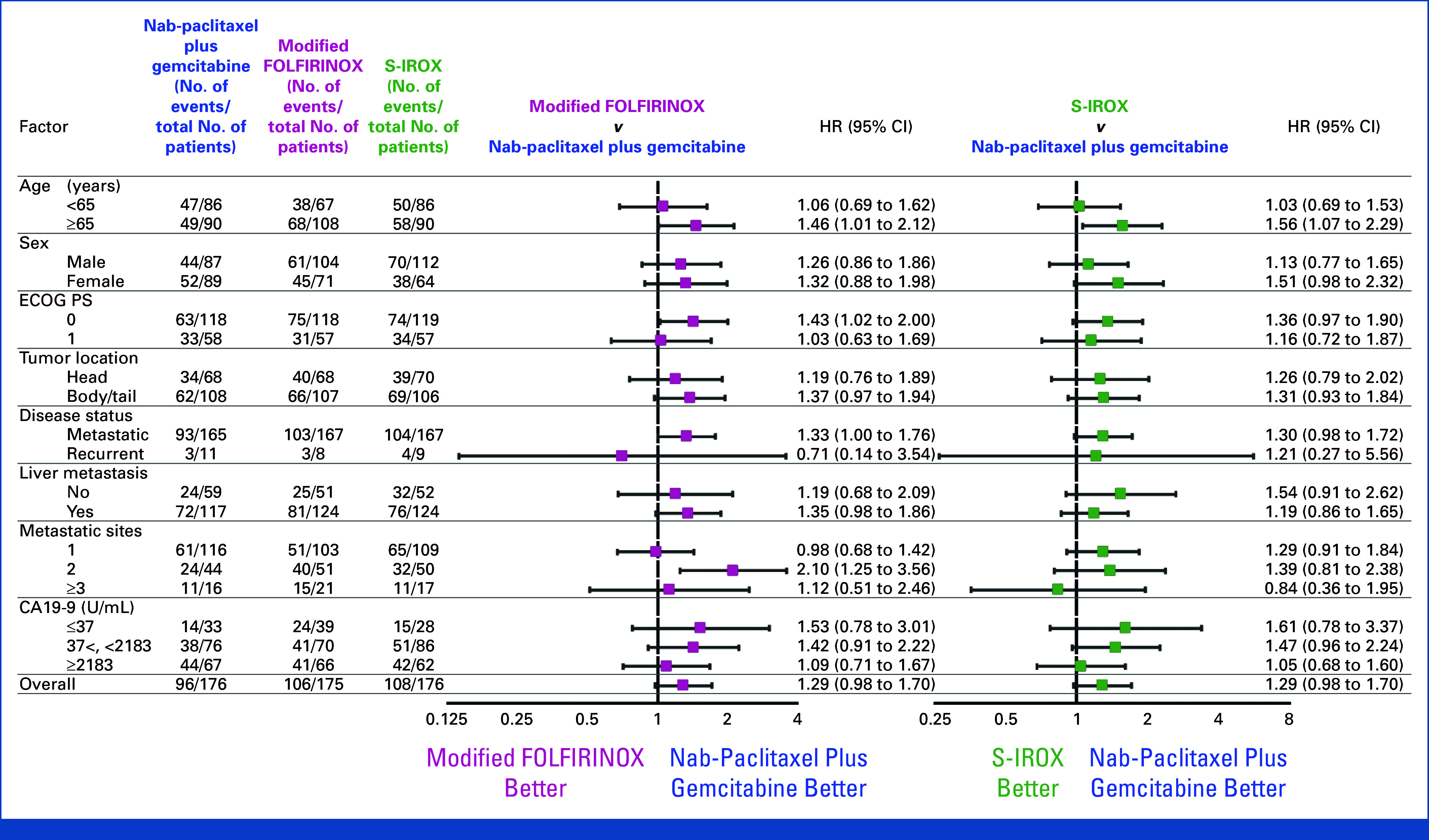
Forest plot of overall survival in selected subgroups. The overall hazard ratio is based on a stratified analysis, and subgroup hazard ratios are based on unstratified analyses. CA19-9, carbohydrate antigen 19-9; ECOG PS, Eastern Cooperative Oncology Group performance status; HR, hazard ratio; mFOLFIRINOX, modified fluorouracil, leucovorin, irinotecan, and oxaliplatin; S-IROX, S-1, irinotecan, and oxaliplatin.

At the data cutoff of the first follow-up survey after the interim analysis, the median duration of follow-up for the ITT population was 10.1 months (IQR, 6.0-17.4). Of the 527 patients, the updated median OS was 17.0 months (95% CI, 14.5 to 18.9) in the nab-paclitaxel + gemcitabine group, 14.0 months (95% CI, 11.4 to 16.3) in the mFOLFIRINOX group (HR, 1.29 [95% CI, 0.98 to 1.70]), and 13.6 months (95% CI, 12.3 to 16.3) in the S-IROX group (HR, 1.29 [95% CI, 0.98 to 1.70]; Fig 2). The OS benefits of nab-paclitaxel + gemcitabine compared with mFOLFIRINOX or S-IROX remained consistent across prespecified subgroups (Fig 3). The median PFS was 6.7 months (95% CI, 5.7 to 7.4) in the nab-paclitaxel + gemcitabine group, 5.8 months (95% CI, 5.1 to 6.9) in the mFOLFIRINOX group (HR, 1.15 [95% CI, 0.91 to 1.45]), and 6.7 months (95% CI, 5.7 to 8.3) in the S-IROX group (HR, 1.07 [95% CI, 0.84 to 1.35]; Fig 2).

Until Mar 2020, 139 patients were enrolled and 46 eligible patients were allocated to the S-IROX group during the phase II portion of the trial. As of Jun 15, 2020, 14 of the 46 patients (30.4%; 80% CI, 21.5 to 40.8) in the S-IROX group achieved a confirmed complete or partial response. As the primary end point of the phase II portion was met, the phase III portion continued as a three-arm trial (Data Supplement, Table S2). The ORRs in the phase III portion were 35.4% (95% CI, 28.4 to 43.0) in the nab-paclitaxel + gemcitabine group, 32.4% (95% CI, 25.4 to 39.9) in the mFOLFIRINOX group, and 42.4% (95% CI, 34.8 to 50.2) in the S-IROX group (Table 2).

**TABLE 2.** Objective Response Rate and Disease Control Rate (patients with measurable lesions)

Best Response	Nab-Paclitaxel + Gemcitabine (n = 175)	mFOLFIRINOX (n = 170)	S-IROX (n = 170)
Objective response rate, % (95% CI)	35.4% (28.4 to 43.0)	32.4% (25.4 to 39.9)	42.4% (34.8 to 50.2)
Disease control rate, % (95% CI)	83.4% (77.1 to 88.6)	72.9% (65.6 to 79.5)	81.8% (75.1 to 87.3)
Best objective response, No. (%)			
Complete response	0 (0)	1 (0.6)	0 (0)
Partial response	62 (35.4)	54 (31.8)	72 (42.4)
Stable disease	84 (48.0)	69 (40.6)	67 (39.4)
Progressive disease	20 (11.4)	31 (18.2)	21 (12.4)
Not evaluable	9 (5.1)	15 (8.8)	10 (5.9)

Abbreviations: mFOLFIRINOX = modified fluorouracil, leucovorin, irinotecan, and oxaliplatin; S-IROX, S-1, irinotecan, and oxaliplatin.

### Subsequent Therapy

The protocol treatment was terminated for the following reasons: disease progression in 92, 85, and 84 patients in the nab-paclitaxel + gemcitabine, mFOLFIRINOX, and S-IROX groups, respectively, and adverse events or refusal associated with adverse events in 42, 60, and 52 patients in the nab-paclitaxel + gemcitabine, mFOLFIRINOX, and S-IROX groups, respectively. In the ITT population, 105 patients (59.7%), 111 patients (63.4%), and 110 patients (62.5%) in the nab-paclitaxel + gemcitabine, mFOLFIRINOX, and S-IROX groups received subsequent anticancer therapy (Data Supplement, Table S3). The most common regimens were the fluoropyrimidine-based regimen in the nab-paclitaxel + gemcitabine group (47.6%) and the gemcitabine-based regimen in the mFOLFIRINOX group (67.6%) and the S-IROX group (73.6%). The liposomal irinotecan-based regimen was administered to 34.3% of patients in the nab-paclitaxel + gemcitabine group, 1.8% of patients in the mFOLFIRINOX group, and 0.9% of patients in the S-IROX group.

### Dose Intensity

Among the 519 patients who received protocol treatment, the median relative dose intensity was 68.5% (IQR, 58.4-84.3) for nab-paclitaxel and 75.4% (IQR, 61.8-86.9) for gemcitabine in the nab-paclitaxel + gemcitabine group; 86.4% (IQR, 77.6-94.7) for fluorouracil, 78.9% (IQR, 66.0-91.0) for oxaliplatin, and 74.9% (IQR, 65.8-90.8) for irinotecan in the mFOLFIRINOX group; and 74.0% (IQR, 64.0-83.4) for S-1, 79.4% (IQR, 64.2-92.1) for oxaliplatin, and 75.2% (IQR, 63.2-93.7) for irinotecan in the S-IROX group (Data Supplement, Table S4).

### Safety

Among the grade 3 to 4 adverse events, neutropenia was more common in the nab-paclitaxel + gemcitabine group (60.3%) than in the mFOLFIRINOX (51.5%) and S-IROX (38.7%) groups. However, anorexia and diarrhea were less common in the nab-paclitaxel + gemcitabine group (5.2% and 1.1%, respectively) than in the mFOLFIRINOX group (22.8% and 8.8%, respectively) and S-IROX (27.6% and 23.0%, respectively) group (Table 3). Treatment-related death occurred in only one patient (0.2%), a patient in the S-IROX group.

**TABLE 3.** Adverse Events (safety population)

Adverse Event Preferred Term	Nab-Paclitaxel + Gemcitabine (n = 174), No. (%)			mFOLFIRINOX (n = 171), No. (%)			S-IROX (n = 174), No. (%)		
	Grade 1 to 2	Grade 3	Grade 4	Grade 1 to 2	Grade 3	Grade 4	Grade 1 to 2	Grade 3	Grade 4
Neutropenia	51 (29.3)	79 (45.4)	26 (14.9)	65 (38.0)	49 (28.7)	39 (22.8)	79 (45.4)	37 (21.3)	30 (17.2)
White blood cell decreased	55 (31.6)	52 (29.9)	8 (4.6)	68 (39.8)	29 (17.0)	9 (5.3)	48 (27.6)	21 (12.1)	7 (4.0)
Anemia	118 (67.8)	15 (8.6)	4 (2.3)	112 (65.5)	13 (7.6)	5 (2.9)	101 (58.0)	17 (9.8)	2 (1.1)
Thrombocytopenia	149 (85.6)	6 (3.4)	2 (1.1)	141 (82.5)	7 (4.1)	1 (0.6)	135 (77.6)	13 (7.5)	2 (1.1)
Febrile neutropenia	NA	6 (3.4)	0	NA	15 (8.8)	0	NA	13 (7.5)	0
Neuropathy	90 (51.7)	19 (10.9)	0	76 (44.4)	14 (8.2)	0	88 (50.6)	13 (7.5)	0
Infection	3 (1.7)	19 (10.9)	0	6 (3.5)	28 (16.4)	0	5 (2.9)	14 (8.0)	1 (0.6)
ALT increased	117 (67.2)	18 (10.3)	1 (0.6)	116 (67.8)	18 (10.5)	2 (1.2)	119 (68.4)	20 (11.5)	0
AST increased	113 (64.9)	13 (7.5)	1 (0.6)	122 (71.3)	15 (8.8)	1 (0.6)	136 (78.2)	11 (6.3)	1 (0.6)
Anorexia	76 (43.7)	9 (5.2)	0	86 (50.3)	39 (22.8)	0	81 (46.6)	48 (27.6)	0
Fatigue	86 (49.4)	5 (2.9)	NA	74 (43.3)	7 (4.1)	NA	79 (45.4)	11 (6.3)	NA
Nausea	49 (28.2)	4 (2.3)	NA	76 (44.4)	15 (8.8)	NA	82 (47.1)	18 (10.3)	NA
Diarrhea	41 (23.6)	2 (1.1)	0	74 (43.3)	14 (8.2)	1 (0.6)	67 (38.5)	40 (23.0)	0

NOTE. Data are No. (%). Grade 3 to 4 adverse events in ≥5% of patients in either treatment group were selected.

Abbreviations: mFOLFIRINOX, modified fluorouracil, leucovorin, irinotecan, and oxaliplatin; NA, not applicable; S-IROX, S-1, irinotecan, and oxaliplatin.

## DISCUSSION

GENERATE was unexpectedly terminated early owing to futility in the planned interim analysis. The OS was numerically longer in the nab-paclitaxel + gemcitabine group than in the mFOLFIRINOX and S-IROX groups. On the other hand, PFS was similar among the three groups. Postprogression survival (PPS) was longer in the nab-paclitaxel + gemcitabine group than in the mFOLFIRINOX and S-IROX groups (Data Supplement, Fig S1), and this is likely the cause of the OS difference. A possible explanation for the difference in PPS is that fluoropyrimidine- or liposomal irinotecan–based regimens are more effective than gemcitabine-based regimens as later lines of treatment. Although liposomal irinotecan in combination with fluorouracil and leucovorin was superior to fluorouracil and leucovorin in the NAPOLI-1 trial,^13^ no randomized trial has demonstrated the superiority of nab-paclitaxel + gemcitabine over gemcitabine monotherapy as a second-line treatment. Nab-paclitaxel + gemcitabine after mFOLFIRINOX may not maintain a sufficient dose intensity because of peripheral neuropathy. Another possible explanation is that discontinuation is owing to adverse events, which were frequent in the mFOLFIRINOX and S-IROX groups. GI toxicities and infections, which make patients prone to hospitalization and subsequent treatment discontinuation, were more common in the mFOLFIRINOX and S-IROX groups. Therefore, at the time of second-line treatment, the systemic status might have been different and decline of systemic status may affect the shorter PPS of chemotherapies.

Overall, all three regimens were tolerable and manageable. However, consistent with the history of development,^7,8^ febrile neutropenia and infections were frequently observed in the mFOLFIRINOX group and 114 of 171 patients (66.7%) required a reduced regimen dose. Thinking about the use in our practice, nab-paclitaxel + gemcitabine may be the preferred regimen because it is less toxic than mFOLFIRINOX and S-IROX in Japanese patients.

It is essential to synthesize the results of GENERATE and NAPOLI-3^5^ trials for a comprehensive discussion on metastatic pancreatic cancer treatment. A plausible explanation is that liposomal irinotecan plays a crucial role in pancreatic cancer treatment. After nab-paclitaxel + gemcitabine, liposomal irinotecan is often administered in a second-line setting, and it was used in 34.3% of patients treated with nab-paclitaxel + gemcitabine in GENERATE. The survival benefit observed with liposomal irinotecan in combination with fluorouracil and leucovorin,^13^ in contrast to the modest activity of fluorouracil, leucovorin, and irinotecan in the second-line setting,^14^ indicates that liposomal irinotecan fundamentally differs from unencapsulated irinotecan. Another plausible explanation is that NALIRIFOX benefits from dose reduction optimization. NALIRIFOX was used at doses (50 mg/m^2^ liposomal irinotecan and 60 mg/m^2^ oxaliplatin once every 2 weeks) lower than the typical doses based on its phase I/II trial results.^15^ Intertrial comparisons have limitations; however, discontinuation owing to adverse events was lower with NALIRIFOX (14.1%) than with mFOLFIRINOX (34.3%) and S-IROX (29.5%) in GENERATE. Assuming similar regimens, racial differences in the treatment responses should be considered. This implies that nab-paclitaxel + gemcitabine may be more suitable for Asian patients, whereas FOLFIRINOX-based regimens may be more suitable for Western patients. As NAPOLI-3 included only 4.9% of Asian patients, its efficacy and safety results may not be representative of Asian patients.

In terms of OS, GENERATE showed a survival time of more than 1 year. Considering that the survival time of NAPOLI-3 was <1 year, the results of GENERATE are extremely good. Comparing patient backgrounds, 67% of patients in GENERATE had a PS of 0 and about 60% had one metastatic site; these values were about 40% and 30% in NAPOLI-3, respectively. Furthermore, the proportion of patients receiving secondary treatment also differed, with approximately 60% of patients in GENERATE and approximately 50% of patients in NAPOLI-3.

In addition, 60% of the patients enrolled in this study had pancreatic body or tail cancer. This is a similar ratio to that in the NAPOLI-3 trial, but the actual situation differs as about 60% of pancreatic cancers are considered to be pancreatic head cancer. Patients with pancreatic head cancer often suffer from repeated cholangitis and liver function abnormalities, and their general conditions often deteriorate, which may make it difficult for them to be enrolled in clinical trials. Pancreatic body or tail cancer might have led to a favorable prognostic outcome.

A detailed analysis of the subgroup in the forest plot also shows slight differences in trends for the various factors. The older patients (age 65 or older) with better responses to nab-paclitaxel + gemcitabine is understandable, as toxicity may have been more frequent in the mFOLFIRINOX. Patients with a PS of 1 or more than 3 metastatic sites seem to be in poorer general condition but showed relatively favorable responses to mFOLFIRINOX; this may be due to the limited sample size or to patients enrolled with a PS of 1 or many metastatic sites having been selected more carefully.

Owing to the demonstrated benefits of first-line platinum-containing regimens^16^ and subsequent olaparib maintenance therapy^17^ in patients harboring germline *BRCA1* or *BRCA2* mutations, first-line treatment with a mFOLFIRINOX or S-IROX regimen should be considered. In this trial, germline mutation testing for *BRCA1/2* was performed in ≤50% of the patients and only 23 patients (4.4%) were documented as having deleterious or suspected deleterious germline *BRCA1*/*2* mutations. The randomized phase II PASS-01 trial compared mFOLFIRINOX with nab-paclitaxel + gemcitabine in patients with metastatic pancreatic cancer, excluding germline *BRCA 1/2* and *PALB2* mutations in the United States and Canada. This study reported an early analysis of a short observation period, but the results showed that the hazard ratio for PFS of mFOLFIRINOX compared with nab-paclitaxel + gemcitabine was 1.39 (95% CI, 0.95 to 2.03) and the hazard ratio for OS was 1.66 (95% CI, 1.10 to 2.49), indicating that nab-paclitaxel + gemcitabine might have better results. Therefore, nab-paclitaxel + gemcitabine may show relatively better results when the number of platinum-sensitive patients is low.

This study had several limitations. First, the trial was conducted exclusively in Japanese centers, and the results may not be directly applicable to Western patients. Second, this was a trial that was stopped because of an interim analysis, and the follow-up period was not necessarily long enough. Third, genomic profiles based on tissue and blood samples were not available for all patients.

In conclusion, mFOLFIRINOX or S-IROX did not appear to show superiority compared with nab-paclitaxel + gemcitabine as the first-line treatment for metastatic or recurrent pancreatic cancer.

## Data Availability

Individual participant data that underlie the results reported in this article (after deidentification) will be shared if investigators who submit a proposal to use the data are approved by the Hepatobiliary and Pancreatic Oncology Group of JCOG.

## APPENDIX

**TABLE A1.** List of Investigators

Institution	Principal Investigator
Sapporo-Kosei General Hospital	Keiya Okamura
Hokkaido University Hospital	Yoshito Komatsu
Teine-Keijinkai Hospital	Akio Katanuma
Tohoku University Hospital	Michiaki Unno
Tochigi Cancer Center	Hirofumi Shirakawa
Jichi Medical University	Hironori Yamaguchi
Saitama Cancer Center	Satoshi Shimizu
National Cancer Center Hospital East	Masafumi Ikeda
Chiba Cancer Center	Hiroshi Ishii
Chiba University, Graduate School of Medicine	Naoya Kato
National Cancer Center Hospital	Takuji Okusaka
Kyorin University Faculty of Medicine	Fumio Nagashima
Tokyo Medical University Hospital	Takao Itoi
National Center for Global Health and Medicine (NCGM)	Yasushi Kojima
Tokyo Women's Medical University	Hidekazu Kuramochi
Cancer Institute Hospital of Japanese Foundation for Cancer Research	Naoki Sasahira
Teikyo University School of Medicine	Keiji Sano
Tokai University School of Medicine	Shinichiro Takahashi
St. Marianna University School of Medicine	Yu Sunakawa
Kanagawa Cancer Center	Makoto Ueno
Kitasato University School of Medicine	Yusuke Kumamoto
Yokohama City University Medical Center	Kazuya Sugimori
Niigata Cancer Center Hospital	Kazuhiko Shioji
Toyama University Hospital	Ichiro Yasuda
Kanazawa University School of Medicine	Taro Yamashita
Ishikawa Prefectural Central Hospital	Kunihiro Tsuji
Shizuoka Cancer Center	Kentaro Yamazaki
Aichi Cancer Center Hospital	Kazuo Hara
Kyoto University Hospital	Etsuro Hatano
Osaka University, Graduate School of Medicine	Hidetoshi Eguchi
Kindai University Hospital	Masatoshi Kudo
Osaka International Cancer Institute	Kazuyoshi Ohkawa
National Hospital Organization Osaka National Hospital	Kunihito Gotoh
Kansai Medical University Hospital	Sohei Satoi
Kobe University Graduate School of Medicine	Kazutoshi Tobimatsu
Hyogo Medical University	Seiko Hirono
Hyogo Cancer Center	Ikuya Miki
Wakayama Medical University, School of Medicine	Masayuki Kitano
Yamaguchi University Hospital	Hiroaki Nagano
National Hospital Organization Shikoku Cancer Center	Akinori Asagi
Kochi Health Sciences Center	Takehiro Okabayashi
National Kyushu Cancer Center	Masayuki Furukawa
Kyushu University Hospital	Nao Fujimori
Nagasaki University Hospital	Kazuhiko Nakao
Kagoshima University Hospital	Akio Ido
